# 1612. Correlates of Sustained Viral Suppression among HIV-Positive Adults Receiving Antiretroviral Therapy from Six Secondary Health Facilities in Northern Nigeria.

**DOI:** 10.1093/ofid/ofad500.1447

**Published:** 2023-11-27

**Authors:** F A T I M A H I TSIGA-AHMED, Muhammad S Musa, Abdulwahab K Sulaiman, Rabiu I Jalo, Abdulaziz T Bako, Sahabi K Sulaiman

**Affiliations:** Bayero University, Kano/ Aminu Kano Teaching Hospital, Kano-Nigeria, KANO, Kano, Nigeria; Yobe State University Teaching Hospital, Damaturu, Yobe, Nigeria; Murtala Muhammad Specialist Hospital/Kwanar Dawaki Isolation Center, Kano, Kano, Nigeria; Bayero University, Kano/ Aminu Kano Teaching Hospital, Kano, Kano, Nigeria; Center for Outcomes Research, Houston Methodist., Houston, Texas; Yobe State University Teaching Hospital, Damaturu, Yobe, Nigeria

## Abstract

**Background:**

Sustained viral suppression in HIV is essential to minimize drug resistance, reduce secondary transmission and improve health outcomes necessary to achieve the UNAIDS 95-95-95 strategy. There is dearth of information on viral suppression among adults in Nigeria. Identifying correlates of sustained viral suppression will inform treatment guidelines especially for people at risk and provide recommendations to policy makers.

**Methods:**

We collected sociodemographic and HIV-related data from a cross-section of 730 HIV-positive adults receiving antiretroviral therapy from six secondary health facilities in northern Nigeria, between October 2021 and February 2022. Sustained viral suppression was defined according to the Nigerian National Guideline for HIV Prevention, Treatment and Care as a viral load below the detection threshold of less than 20 copies of HIV RNA/ml. Proportion of participants with sustained viral suppression was estimated and a multivariable analysis used to identify independent correlates of sustained viral suppression.

**Results:**

The sustained viral suppression rate among HIV-positive adults on ART was 35.7% [95% CI: 32.2, 39.5]. Being married [AOR 2.01; 95% CI: 1.34, 3.03] and having secondary education [AOR 0.43; 95% CI: 0.26, 0.74] were significantly associated with sustained viral suppression. Respondents on treatment for 5-10 years [AOR 1.76; 95% CI: 1.17, 2.64] and those taking two or more pills [AOR 5.67; 95% CI: 3.74, 8.62] were more likely to have sustained viral suppression. Previous treatment for opportunistic infection [AOR 0.24; 95% CI: 0.15, 0.41] significantly decreased the likelihood of sustained viral suppression.

**Figure:**
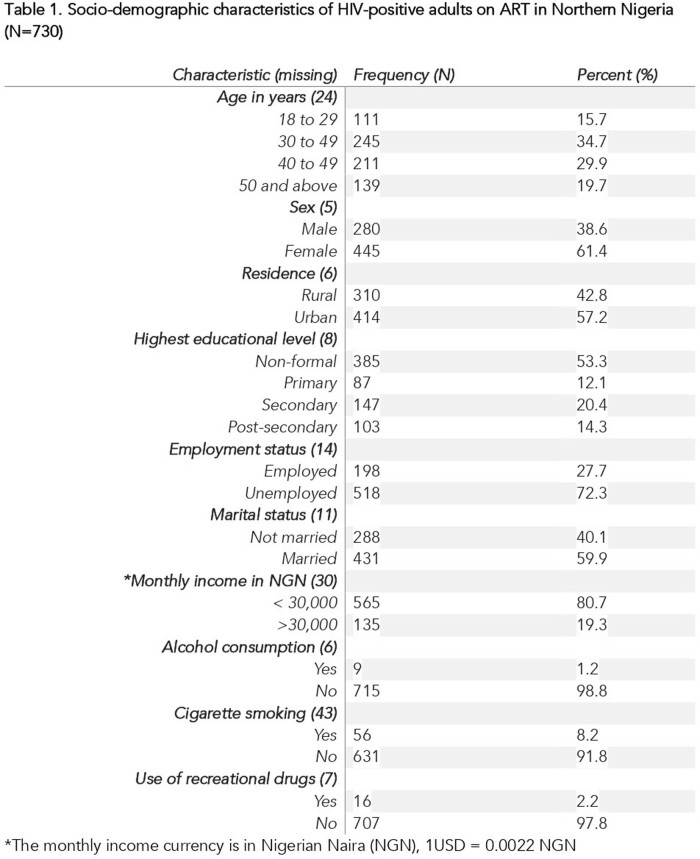

**Figure:**
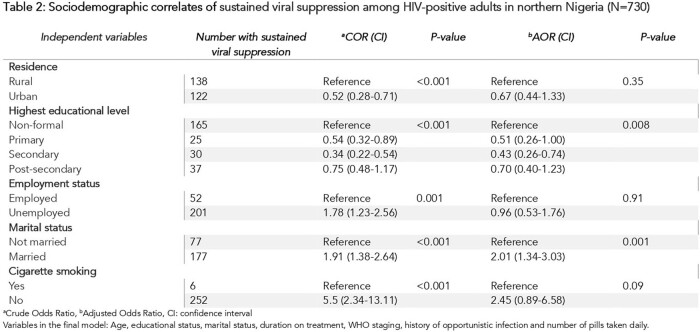

**Figure:**
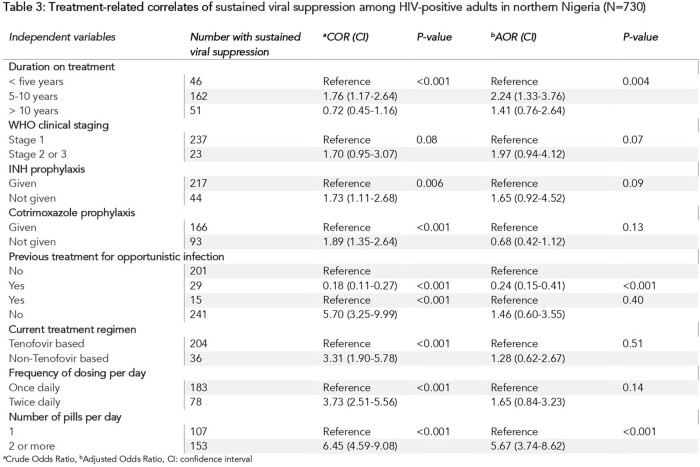

**Conclusion:**

Sustained viral suppression rates among HIV-positive adults on ART in the six facilities were suboptimal. While being married, taking two or more ART pills and being on treatment for 5-10 years increased the likelihood of having a suppressed viral load, being educated up to secondary level and previous treatment for opportunistic infection decreased the odds of having sustained viral suppression. There is a need to improve viral load monitoring and direct viral suppression interventions towards at risk population.

**Disclosures:**

**All Authors**: No reported disclosures

